# P2Y12 Inhibitors in Acute Coronary Syndromes: A Real-World, Community-Based Comparison of Ischemic and Bleeding Outcomes

**DOI:** 10.1155/2023/1147352

**Published:** 2023-05-20

**Authors:** Amit Sachdeva, Ratnabhushan Mutyala, Neha Mantri, Shiyun Zhu, Edward McNulty, Matthew Solomon

**Affiliations:** ^1^Division of Cardiology, Kaiser Permanente Northern California, Walnut Creek, California, USA; ^2^Drexel University College of Medicine, Philadelphia, Pennsylvania, USA; ^3^Division of Cardiology, Kaiser Permanente Northern California, San Francisco, California, USA; ^4^Division of Research, Kaiser Permanente Northern California, Oakland, California, USA; ^5^Division of Cardiology, Kaiser Permanente Northern California, Oakland, California, USA

## Abstract

**Background:**

Randomized trials have shown superiority of the novel P2Y12 inhibitors over clopidogrel in patients with acute coronary syndrome (ACS), but clinical benefit in the community remains controversial. Our objective was to compare the safety and efficacy of clopidogrel to ticagrelor and prasugrel in patients with ACS undergoing percutaneous coronary intervention (PCI) in a real-world population.

**Methods:**

We conducted a retrospective cohort study of patients with ACS who underwent PCI and were discharged with clopidogrel, ticagrelor, or prasugrel from 2012 to 2018 within Kaiser Permanente Northern California. We used Cox proportional hazard models with propensity-score matching to evaluate the association of the P2Y12 agent with the primary outcomes of all-cause mortality, myocardial infarction (MI), stroke, and bleeding events.

**Results:**

The study included 15,476 patients (93.1% on clopidogrel, 3.6% on ticagrelor and 3.2% on prasugrel). Compared to the clopidogrel group, ticagrelorand prasugrel patients were younger with less comorbidities. In multivariable models with propensity-score matching, we found a lower risk of all-cause mortality in the ticagrelor vs the clopidogrel group (HR (95% CI) 0.43 (0.20–0.92)), but no differences in the other endpoints, and no difference between prasugrel and clopidogrel among any endpoints. A larger proportion of patients on ticagrelor or prasugrel switched to an alternative P2Y12 agent vs. clopidogrel (*p* < 0.01), and a higher level of persistence was seen among patients on clopidogrel vs. ticagrelor (*p* = 0.03) or prasugrel (*p* < 0.01).

**Conclusion:**

Among patients with ACS who underwent PCI, we observed a lower risk of all-cause mortality in patients treated with ticagrelor vs clopidogrel, but no difference in other clinical endpoints nor any differences in endpoints between prasugrel vs. clopidogrel users. These results suggest that further study is needed to identify an optimal P2Y12 inhibitor in a real-world population.

## 1. Introduction

Randomized trial data have demonstrated an approximately 2% reduction in ischemic events and a 0.5–1% increase in bleeding events with ticagrelor or prasugrel versus clopidogrel in those presenting with acute coronary syndrome (ACS) [1, 2]. These data have not been uniformly replicated in real-world, nonclinical trial populations [3–5]. The largest of these studies evaluated ticagrelor versus clopidogrel and showed that 1 year risk of net adverse clinical events was not different between the groups after propensity score matching, with higher rates of bleeding and dyspnea in the ticagrelor group [3]. In this study, we aimed to determine the comparative efficacy and safety of the novel P2Y12 inhibitors versus clopidogrel in a large integrated healthcare delivery system, among those presenting with ACS undergoing percutaneous coronary intervention (PCI).

## 2. Methods

### 2.1. Study Population

Kaiser Permanente Northern California (KPNC) is an integrated healthcare delivery system providing comprehensive care to more than 4.5 million members in Northern California. The health plan owns and operates 21 medical centers, including >250 ambulatory care facilities, pharmacies, and laboratories, providing comprehensive inpatient, emergency department and outpatient care, with nearly all care captured through its electronic health record (EHR) system that is integrated across all practice settings. Members of KPNC are broadly representative of the California population in terms of ethnic and socioeconomic profile [6]. The study population included patients who underwent PCI for ACS within the twelve regional cardiac catheterization laboratories of KPNC between January 1st, 2012, and December 31st, 2018. This start date was chosen because ticagrelor, which is the newer of the two novel agents, was FDA-approved in 2011. The study was approved by the KPNC institutional review board with a waiver of consent.

**Figure 1:**
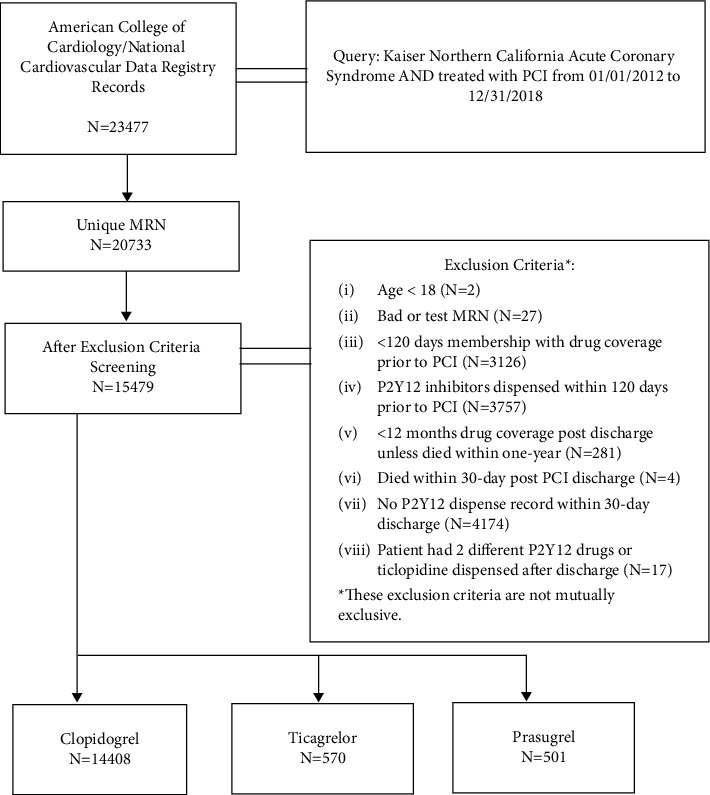
Consort diagram of cohort assembly.

ACS PCI cases were initially identified from KPNC's cardiac catheterization labs' American College of Cardiology/National Cardiovascular Data Registry (ACC/NCDR) database [7]. Exclusion criteria included (1) less than 18 years of age at the time of PCI, (2) less than 120 days membership with drug coverage prior to PCI, (3) P2Y12 inhibitors dispensed within 120 days prior to PCI, (4) less than 12 months membership with drug coverage post PCI discharge unless died within one year, (5) death within 30 days post PCI discharge, (6) no P2Y12 inhibitor dispense record within 30 days after discharge, (7) two different P2Y12 drugs dispensed on the same day postdischarge, and (8) patients who were prescribed with ticlopidine (Figure 1). Index event was assigned as the first qualified hospitalized ACS event during the study period.

### 2.2. Exposure

P2Y12 antagonist use was obtained electronically, using previously validated methods [8]. Briefly, patients were determined to be taking one of the three P2Y12 antagonists if they had dispense records of ticagrelor, prasugrel, or clopidogrel within 30-days post index PCI discharge.

Adherence, persistence, and switching of P2Y12 inhibitors were assessed using previously defined methods [4]. Adherence, or medication refill adherence (MRA), was defined as the total days of medication supply in one year divided by 365 and was expressed as a percentage. Patients with an MRA >80% were defined as adherent. We considered patients “nonpersistent” if the gap between refills exceeded the days of supply plus a 15-day grace period. Drug switching was defined as more than one P2Y12 inhibitor within the first 365 days. For subjects who switched P2Y12 inhibitors during the study, all P2Y12 fill information was considered for the calculation of adherence and persistence (i.e., patients were considered adherent if they filled the second P2Y12 inhibitor at appropriate intervals).

### 2.3. Outcome

The primary study outcomes included all-cause mortality, hospitalized myocardial infarction, hospitalized stroke, and hospitalized bleeding events examined within the first year after the index ACS event. All hospitalized events were determined using ICD codes with principal, primary, or secondary diagnoses during the hospitalization. Patients were followed from the index event until they died, had an outcome of interest, or for at most one year, whichever occurred first. All hospitalized outcomes were collected independently (e.g., patients who had hospitalized stroke continued to be followed for other outcomes).

### 2.4. Data Collection

Baseline demographic, laboratory data, procedural data, and medication use after index PCI were extracted from various KPNC electronic databases including variables submitted to the ACC/NCDR Cath PCI Quality Registry in accordance with definitions specified in both version 4.4 and 5 [7]. Baseline comorbidities were identified using ICD-9 and 10 code definitions within one-year prior to the index event. Laboratory data were obtained at the time of cardiac catheterization or the most recent value within one day before the procedure. The use of other cardiac medications was identified within 30-days post index event. Additionally, the PRECISE DAPT score, a validated score for predicting bleeding risk after stent implantation, was calculated for each patient [9].

### 2.5. Statistical Analysis

Descriptive statistics were used to describe the demographic and clinical characteristics of the cohort by P2Y12 inhibitor use. We used clopidogrel as our reference group and ticagrelor or prasugrel was compared to clopidogrel, respectively. Differences in characteristics were assessed using the two-sample *t*-tests for continuous variables and the chi-square tests for categorical variables. These tests were also used to assess differences in adherence, persistence, and drug switch between novel P2Y12 inhibitors and clopidogrel from index event to one-year follow-up. Trends in P2Y12 inhibitor use during the study period were evaluated using the Cochran–Mantel–Haenszel test.

We used the Kaplan–Meier (KM) method to analyze outcomes in patients across one-year of follow-up. Comparisons between patients on different P2Y12 inhibitors were conducted using log-rank tests. We calculated the hazard ratio (HR) and 95% confidence interval (CI) for the associations between P2Y12 inhibitor use and each outcome using bivariate and multivariable Cox proportional hazards (PH) regression models. Covariates for the multivariable models were selected a priori based on previously published studies, clinical relevance, or a *p* value <0.1 from bivariate analyses assessing demographic and clinical characteristics and P2Y12 inhibitor use. We performed two steps of adjustment by including all potential covariates in the multivariable models first, and subsequently dropped those covariates that did not have significant associations (i.e., *p* > 0.05) with the outcomes except demographic characteristics and medication adherence rate, based upon a priori relevance to the research question.

Since the choice of P2Y12 inhibitor may be associated with other prognostic factors, we also performed propensity score (PS) matching using the optimal variable ratio matching method to balance the groups at baseline. Each patient in the ticagrelor or prasugrel group was matched with patients in the clopidogrel group with minimal 1 : 1 match and maximal 1 : 5 match. Caliper width was 0.25. Baseline variables selected for PSM included age, gender, and all clinical risk factors demonstrated *p* < 0.1 in Table 1 (i.e., all cardiovascular history and risk factors, smoking status, chronic kidney disease, chronic lung disease, and PCI indication). These variables are also controlled in the subsequent multivariable models, with additional variables that are deemed statistically significant in unadjusted analysis or clinically important. This study was presented as an abstract at the Society for Cardiovascular Angiography and Interventions (SCAI) conference in May, 2022 [10]. During the preparation of the manuscript, it was realized that hypertension was inadvertently omitted from the statistical models (both multivariate and propensity matching) for the abstract. Given that hypertension is an important risk factor that should be adjusted for, we made sure it was added into the models and the analysis was rerun—this explains the results on the manuscript that are discordant from the abstract.

All data extraction and analyses were performed using SAS 9.4 (Cary, North Carolina).

## 3. Results

**Figure 2:**
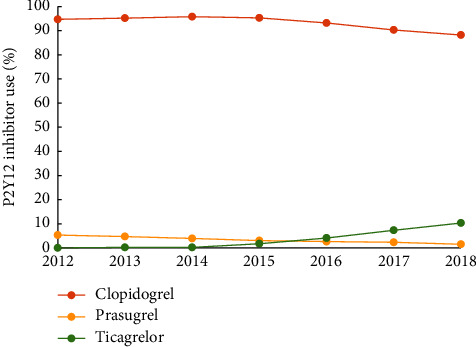
Changes in P2Y12 inhibitor use through the study period between 2021 and 2018. Trends over time was evaluated using the Cochran–Mantel–Haenszel test with *p* < 0.001.

We identified 15,479 patients who satisfied all inclusion and exclusion criteria. Of these, 93% were dispensed with clopidogrel, 3.6% ticagrelor, and 3.2% prasugrel. Clopidogrel use dropped over time from 94.7% of total dispensed in 2012 to 88.2% in 2018, while ticagrelor use increased from 0% to 10.2%, and prasugrel use decreased from 5.3% to 1.5% (*p* < 0.001) (Figure 2).

The mean age at index PCI was 66.3 (standard deviation [SD] ± 12.0) years and 27.8% of the cohort were female (Table 1). Compared to the ticagrelor or prasugrel groups, the clopidogrel group was older, had a higher proportion of female patients, was more likely to have a significant cardiovascular history such as prior CABG, cerebrovascular disease, and heart failure, and had more comorbidities such as chronic kidney disease and hypertension (all *p* < 0.05). Additionally, the clopidogrel group had lower hemoglobin and platelet values compared to either the ticagrelor or prasugrel group (*p* < 0.001). A larger proportion of patients in the prasugrel (40.7%) or ticagrelor (49.8%) group had ST-elevation myocardial infarction (STEMI) as their PCI indication versus clopidogrel (20%), and a higher proportion of these patients were classified as emergency cases and having cardiogenic shock. Clopidogrel patients also had more veins graft PCI, as well as higher PRECISE DAPT scores, and higher use of concomitant oral anticoagulation.  Table 2 shows the baseline characteristics of the propensity-matched population, and Supplementary Tables 1a and 1b demonstrate the characteristics of the clopidogrel patients that were excluded in propensity matching with ticagrelor and prasugrel, respectively.

Adherence to P2Y12 inhibitor therapy was similar across the three groups (Table 3). However, a higher proportion of patients on novel P2Y12 inhibitors switched drugs during follow-up and most of these (92%) switched to clopidogrel. Additionally, persistence to treatment was lower in the prasugrel and ticagrelor group, compared to the clopidogrel group (*p* < 0.03 for ticagrelor vs clopidogrel; *p* < 0.001 for prasugrel vs clopidogrel).

At one-year follow-up, the clopidogrel group had higher mortality rates than the ticagrelor group (*p* < 0.01) (Table 4). The incidence of myocardial infarction, stroke, and bleeding events were similar across the three groups. The Kaplan–Meier curves for the outcomes are included in Supplementary Figure 1. Table 5 shows the incidence of adverse events in the propensity-matched populations.

After combined propensity and multivariable adjustment, ticagrelor was associated with a lower risk of all-cause mortality, compared to clopidogrel (adjusted HR 0.43, 95% CI, 0.20–0.92) (Table 6). There were no differences in myocardial infarction, stroke, or bleeding events between the ticagrelor and clopidogrel groups. Similarly, after adjustment, there were no differences between clopidogrel and prasugrel groups in risks of death, myocardial infarction, stroke, or bleeding events.

## 4. Discussion

After combined multivariable and propensity adjustment in patients undergoing PCI for ACS, the use of ticagrelor compared to clopidogrel was associated with a lower risk of all-cause mortality, but similar rates of hospitalized myocardial infarction, stroke, and bleeding. We found no differences between prasugrel and clopidogrel for any of the adverse outcomes.

In the PLATO trial, the pivotal randomized trial that compared ticagrelor with clopidogrel in ACS, investigators found a similar lower hazard for all-cause death with ticagrelor use, but this was also accompanied by lower rates of myocardial infarction and death from vascular causes [1]. In our study, without any differences in the rates of myocardial infarction or stroke, it is difficult to ascertain a mechanism for associated differences in all-cause mortality. Several similar observational studies have failed to show an association between novel P2Y12 inhibitors and myocardial infarction despite its demonstration in randomized trials, while others have replicated some of the trial findings [3–5, 11–14].

Randomized trials, the gold standard for isolating a treatment effect while minimizing bias, create rarefied environments that are not necessarily reflective of daily patient care. For example, the PLATO trial excluded patients on oral anticoagulation, dialysis patients, clinically important thrombocytopenia and anemia, as well as “any other condition that may put the patient at risk or influence study results in the investigators' opinion (e.g., cardiogenic shock, severe hemodynamic instability, active cancer).” In addition, any condition that increases the risk of medication noncompliance or being lost to follow-up was excluded.

The TRITON TIMI 38 trial, [2] which led to the approval of prasugrel in ACS patients undergoing PCI, similarly has a number of exclusion criteria, including, but not limited to, cardiogenic shock, NYHA class IV congestive heart failure, “clinical findings, in the judgement of the investigator, associated with an increased risk of bleeding,” history of hemorrhagic stroke, ischemic stroke within 3 months, platelet count less than 100,000, anemia (hemoglobin <10 g/dL) at the time of screening, presence of concomitant oral anticoagulation, known severe hepatic dysfunction, and “concomitant medical illness that in the opinion of the investigator is associated with reduced survival over the expected treatment period.”

These protocols exclude many important patient phenotypes. For example, in our study, there were 218 patients with cardiogenic shock, 3201 patients with heart failure, 1136 with liver disease, 420 on dialysis, and 1563 on oral anticoagulation. There also tends to be less diversity in ethnic representation in randomized trials, with >90% of the participants in both PLATO and TRITON TIMI 38 being white, compared to over one-third of our patient population being nonwhite.

A higher proportion of patients in the ticagrelor and prasugrel groups switched drugs compared to the clopidogrel group, and most of them switched to clopidogrel. Both ticagrelor and prasugrel have higher cost [15], and thus that may have played a role, and there is a potential risk of stent thrombosis if switching is not done properly early after stent placement [16]. Also, a higher proportion of patients on clopidogrel were persistent to treatment compared with ticagrelor and prasugrel, which is an observation that has been made before [17] and which also has implications for treatment effect, as premature antiplatelet discontinuation has been identified as the single most important predictor of stent thrombosis [18].

Our study has a number of limitations. It was an observational study, and P2Y12 inhibitor treatment was not randomly assigned. Any differences or lack of differences shown in our study could still be influenced by unmeasured confounders. The larger sample size of clopidogrel patients compared to ticagrelor and prasugrel may limit the statistical power of the analysis. Also, aspirin use was not available through the pharmacy dispense database.

Ascertainment of exposure, namely, P2Y12 inhibitor, was based on pharmacy dispense records. Pill counts may have provided a more precise estimate of thienopyridine use, but the use of filled prescriptions has been shown to reflect actual medication use by patients with a high degree of accuracy [19].

## 5. Conclusion

In patients undergoing PCI for acute coronary syndrome, after both multivariable and propensity adjustment, ticagrelor compared to clopidogrel was associated with lower all-cause mortality, and yet similar rates of myocardial infarction, stroke, and bleeding, while prasugrel compared to clopidogrel was associated with similar rates of all studied, clinically important outcomes. In addition, there was more switching of therapy in patients initially receiving novel P2Y12 inhibitors and these patients tended to be less persistent to treatment. Additional randomized studies, and ones that are more inclusive of patients in real-world practice, are needed to clarify these effects.

## Figures and Tables

**Table 1:** Baseline characteristics, ticagrelor, and prasugrel compared to clopidogrel.

Characteristics	Clopidogrel (*n* = 14407)	Ticagrelor (*n* = 568)	*p*	Prasugrel (*n* = 501)	*p*
*Demographics*					
Age	66.5 ± 12.0	61.3 ± 11.7	<0.001	58.1 ± 10.5	<0.001
Female gender	4036 (28.0)	130 (22.9)	0.01	97 (19.4)	<0.001
Race			0.03		0.09
White	9592 (66.6)	378 (66.5)		318 (63.5)	
Black	866 (6.0)	20 (3.5)		28 (5.6)	
Asian	2542 (17.6)	101 (17.8)		89 (17.8)	
Other/unknown	1407 (9.8)	69 (12.2)		66 (13.1)	
Hispanic ethnicity	1813 (12.6)	378 (66.5)	0.35	69 (13.8)	0.43
*Cardiovascular history*					
Prior MI	3161 (21.9)	86 (15.1)	<0.001	117 (23.4)	0.45
Prior CABG	1627 (11.3)	30 (5.3)	<0.001	30 (6.0)	<0.001
Cerebrovascular disease	1220 (8.5)	25 (4.4)	0.001	7 (1.4)	<0.001
Peripheral vascular disease	5573 (38.7)	137 (24.1)	<0.001	105 (21.0)	<0.001
Heart failure	3121 (21.7)	80 (14.1)	<0.001	76 (15.2)	<0.001
Atrial fibrillation	1618 (11.2)	23 (4.0)	<0.001	22 (4.4)	<0.001
Hyperlipidemia	9889 (68.6)	305 (53.7)	<0.001	281 (56.1)	<0.001
Hypertension	10828 (75.2)	358 (63.0)	<0.001	321 (64.1)	<0.001
Diabetes	5069 (35.2)	176 (31.0)	0.04	137 (27.3)	<0.001
*Other risk factors*					
Smoking			<0.001		<0.001
Previous	5632 (39.1)	166 (29.2)		166 (33.1)	
Current	1848 (12.8)	88 (15.5)		95 (19.0)	
No/unknown	6927 (48.1)	314 (55.3)		240 (47.9)	
Chronic kidney disease	3627 (25.2)	86 (15.1)	<0.001	74 (14.8)	<0.001
Chronic lung disease	1429 (9.9)	44 (7.7)	0.09	36 (7.2)	0.04
Dialysis	412 (2.9)	8 (1.4)	0.04	5 (1.0)	0.01
Dyslipidemia	11380 (79.0)	401 (70.6)	<0.001	362 (72.3)	<0.001
Family history of premature CAD	1888 (13.1)	87 (15.3)	0.13	93 (18.6)	<0.001
Liver disease	1101 (7.6)	35 (6.2)	0.19	37 (7.4)	0.83
*Laboratory data*					
Creatinine	1.1 ± 1.0	1.1 ± 1.0	0.84	1.0 ± 0.7	<0.001
Hemoglobin	13.4 ± 1.9	14.3 ± 1.8	<0.001	14.1 ± 1.8	<0.001
Platelets	216.1 ± 68.9	234.8 ± 65.3	<0.001	226.5 ± 79.9	0.01
Sodium	138.4 ± 3.2	138.0 ± 3.0	0.01	139.1 ± 3.2	<0.001
WBC count	8.6 ± 3.7	9.6 ± 3.1	<0.001	9.4 ± 3.4	<0.001
*Procedure details*					
PCI indication			<0.001		<0.001
NSTEMI/unstable angina	11520 (80.0)	285 (50.2)		297 (59.3)	
STEMI	2887 (20.0)	283 (49.8)		204 (40.7)	
Bare metal stent	685 (4.8)	8 (1.4)	<0.001	19 (3.8)	0.32
Drug eluting stent	13149 (91.3)	543 (95.6)	<0.001	469 (93.6)	0.07
Thrombectomy	807 (5.6)	33 (5.8)	0.83	70 (14.0)	<0.001
Bifurcation lesion	3123 (21.7)	54 (9.5)	<0.001	99 (19.8)	0.31
Lesion complexity			0.49		0.06
High/C	7643 (53.1)	293 (51.6)		287 (57.3)	
Nonhigh/non-C	6764 (46.9)	275 (48.4)		214 (42.7)	
Previously treated lesion	750 (5.2)	25 (4.4)	0.40	53 (10.6)	<0.001
Vein graft PCI	594 (4.1)	12 (2.1)	0.02	12 (2.4)	0.05
Cardiac arrest within 24 hours	226 (1.6)	12 (2.1)	0.31	17 (3.4)	0.002
Cardiomyopathy or LV systolic dysfunction	1700 (11.8)	18 (3.2)	<0.001	36 (7.2)	0.002
Cardiogenic shock within 24 hours	206 (1.6)	12 (3.2)	0.01	14 (2.9)	0.02
Stress test	3511 (24.4)	84 (14.8)	<0.001	100 (20.0)	0.02
Arterial access site			<0.001		<0.001
Brachial	17 (0.1)	0 (0.0)		1 (0.2)	
Femoral	8049 (55.9)	125 (22.0)		335 (66.9)	
Radial	6334 (44.0)	442 (77.8)		165 (32.9)	
Other	7 (0.0)	1 (0.2)		0 (0.0)	
Fluoroscopy time	17.7 ± 13.1	16.8 ± 11.9	0.07	18.7 ± 15.0	0.12
Contrast volume (ml)	178.9 ± 82.0	163.6 ± 67.1	<0.001	213.1 ± 99.3	<0.001
Lesion length	29.3 ± 21.3	31.2 ± 20.2	0.04	31.1 ± 23.0	0.08
*Risk score*					
Precise DAPT score	27.7 ± 19.3	18.4 ± 13.6	<0.001	18.7 ± 16.1	<0.001
*Medication use post PCI*					
ACE inhibitors	8872 (61.6)	406 (71.5)	<0.001	351 (70.1)	<0.001
ARBs	3680 (25.5)	139 (24.5)	0.57	101 (20.2)	0.01
Oral anticoagulation	1541 (10.7)	22 (3.9)	<0.001	30 (6.0)	<0.001
Beta blockers	13532 (93.9)	542 (95.4)	0.14	477 (95.2)	0.24
Statins	13983 (97.1)	557 (98.1)	0.16	490 (97.8)	0.33

MI, myocardial infarction; CABG, coronary artery bypass graft; CAD, coronary artery disease; WBC, white blood cell; NSTEMI, non-ST-elevation myocardial infarction; STEMI, ST-elevation myocardial infarction; ACE inhibitors, angiotensin-converting enzyme inhibitors; and ARBs, angiotensin II receptor blockers.

**Table 2:** Baseline characteristics after propensity matching.

Characteristics	Clopidogrel	Ticagrelor	*p*	Clopidogrel	Prasugrel	*p*
	(*n* = 1704)	(*n* = 568)		(*n* = 1503)	(*n* = 501)	
*Demographics*						
Age	62.1 ± 10.8	61.3 ± 11.7	0.13	60.3 ± 10.3	58.1 ± 10.5	<0.001
Female gender	313 (18.4)	130 (22.9)	0.02	229 (15.2)	97 (19.4)	0.03
Race			0.82			0.44
White	1097 (64.4)	378 (66.5)		944 (62.8)	318 (63.5)	
Black	99 (5.8)	20 (3.5)		85 (5.7)	28 (5.6)	
Asian	319 (18.7)	101 (17.8)		293 (19.5)	89 (17.8)	
Other/unknown	189 (11.1)	69 (12.2)		181 (12.0)	66 (13.1)	
Hispanic ethnicity	213 (12.5)	64 (11.3)	0.44	190 (12.6)	69 (13.8)	0.51
*Cardiovascular history*						
Prior MI	209 (12.3)	86 (15.1)	0.08	257 (17.1)	117 (23.4)	0.002
Prior CABG	92 (5.4)	30 (5.3)	0.91	83 (5.5)	30 (6.0)	0.70
Cerebrovascular disease	55 (3.2)	25 (4.4)	0.19	21 (1.4)	7 (1.4)	1.00
Peripheral vascular	379 (22.2)	137 (24.1)	0.35	316 (21.0)	105 (21.0)	0.97
*Disease*						
Heart failure	204 (12.0)	80 (14.1)	0.19	182 (12.1)	76 (15.2)	0.08
Atrial fibrillation	66 (3.9)	23 (4.0)	0.85	57 (3.8)	22 (4.4)	0.55
Hyperlipidemia	913 (53.6)	305 (53.7)	0.96	796 (53.0)	281 (56.1)	0.22
Hypertension	1048 (61.5)	358 (63.0)	0.52	940 (62.5)	321 (64.1)	0.54
Diabetes	435 (25.5)	176 (31.0)	0.01	381 (25.3)	137 (27.3)	0.38
*Other risk factors*						
Smoking			0.52			0.87
Previous	532 (31.2)	166 (29.2)		493 (32.8)	166 (33.1)	
Current	237 (13.9)	88 (15.5)		272 (18.1)	95 (19.0)	
No/unknown	935 (54.9)	314 (55.3)		738 (49.1)	240 (47.9)	
Chronic kidney disease	235 (13.8)	86 (15.1)	0.42	206 (13.7)	74 (14.8)	0.55
Chronic lung disease	96 (5.6)	44 (7.7)	0.07	95 (6.3)	36 (7.2)	0.50
Dialysis	28 (1.6)	8 (1.4)	0.70	24 (1.6)	5 (1.0)	0.33
Dyslipidemia	1182 (69.4)	401 (70.6)	0.58	1063 (70.7)	362 (72.3)	0.51
Family history of premature CAD	247 (14.5)	87 (15.3)	0.63	230 (15.3)	93 (18.6)	0.09
Liver disease	117 (6.9)	35 (6.2)	0.56	92 (6.1)	37 (7.4)	0.32
*Laboratory data*						
Creatinine	1.0 ± 0.8	1.1 ± 1.0	0.08	1.0 ± 0.9	1.0 ± 0.7	0.56
Hemoglobin	14.0 ± 1.8	14.3 ± 1.8	<0.001	14.0 ± 1.7	14.1 ± 1.8	0.15
Platelets	220.3 ± 68.9	234.8 ± 65.3	<0.001	223.0 ± 70.9	226.5 ± 79.9	0.41
Sodium	138.6 ± 3.0	138.0 ± 3.0	<0.001	138.7 ± 2.9	139.1 ± 3.2	0.01
WBC count	9.0 ± 3.6	9.6 ± 3.1	<0.001	8.9 ± 3.2	9.4 ± 3.4	0.01
*Procedure details*						
PCI indication			<0.001			<0.001
NSTEMI/unstable angina	1183 (69.4)	285 (50.2)		1070 (71.2)	297 (59.3)	
STEMI	521 (30.6)	283 (49.8)		433 (28.8)	204 (40.7)	
Bare metal stent	90 (5.3)	8 (1.4)	<0.001	53 (3.5)	19 (3.8)	0.78
Drug eluting stent	1557 (91.4)	543 (95.6)	0.001	1389 (92.4)	469 (93.6)	0.37
Thrombectomy	128 (7.5)	33 (5.8)	0.17	96 (6.4)	70 (14.0)	<0.001
Bifurcation lesion	386 (22.7)	54 (9.5)	<0.001	309 (20.6)	99 (19.8)	0.70
Lesion complexity			0.08			0.18
High/C	950 (55.8)	293 (51.6)		809 (53.8)	287 (57.3)	
Nonhigh/non-C	754 (44.2)	275 (48.4)		694 (46.2)	214 (42.7)	
Previously treated lesion	61 (3.6)	25 (4.4)	0.37	68 (4.5)	53 (10.6)	<0.001
Vein graft PCI	36 (2.1)	12 (2.1)	1.00	32 (2.1)	12 (2.4)	0.72
Cardiac arrest within 24 hours	38 (2.2)	12 (2.1)	0.87	30 (2.0)	17 (3.4)	0.07
Cardiomyopathy or LV systolic dysfunction	147 (8.6)	18 (3.2)	<0.001	121 (8.1)	36 (7.2)	0.53
Cardiogenic shock within 24 hours	29 (1.9)	12 (3.2)	0.11	20 (1.5)	14 (2.9)	0.04
Stress test	391 (22.9)	84 (14.8)	<0.001	313 (20.8)	100 (20.0)	0.68
Arterial access site			<0.001			<0.001
Brachial	4 (0.2)	0 (0.0)		2 (0.1)	1 (0.2)	
Femoral	872 (51.2)	125 (22.0)		761 (50.6)	335 (66.9)	
Radial	1 (0.1)	1 (0.2)		2 (0.1)	165 (32.9)	
Other	827 (48.5)	442 (77.8)		738 (49.1)	0 (0.0)	
Fluoroscopy time	16.8 ± 12.6	16.8 ± 11.9	0.95	16.6 ± 12.2	18.7 ± 15.0	0.004
Contrast volume (ml)	181.6 ± 78.7	163.6 ± 67.1	<0.001	181.0 ± 81.4	213.1 ± 99.3	<0.001
Lesion length	29.4 ± 20.7	31.2 ± 20.2	0.07	29.4 ± 21.2	31.1 ± 23.0	0.14
*Risk score*						
Precise DAPT score	21.4 ± 16.2	18.6 ± 13.5	<0.001	20.7 ± 15.7	19.3 ± 15.6	0.09
*Medication use post PCI*						
ACE inhibitors	1121 (65.8)	406 (71.5)	0.01	1028 (68.4)	351 (70.1)	0.49
ARBs	388 (22.8)	139 (24.5)	0.41	306 (20.4)	101 (20.2)	0.92
Oral anticoagulation	100 (5.9)	22 (3.9)	0.07	90 (6.0)	30 (6.0)	1.00
Beta blockers	1619 (95.0)	542 (95.4)	0.69	1436 (95.5)	477 (95.2)	0.76
Statins	1673 (98.2)	557 (98.1)	0.86	1483 (98.7)	490 (97.8)	0.17

MI, myocardial infarction; CABG, coronary artery bypass graft; CAD, coronary artery disease; WBC, white blood cell; NSTEMI, non-ST-elevation myocardial infarction; STEMI, ST-elevation myocardial infarction; ACE inhibitors, angiotensin-converting enzyme inhibitors; and ARBs, angiotensin II receptor blockers.

**Table 3:** Changes in medication use from index to 12-month follow-up.

Adherence	Clopidogrel (*N* = 14408)	Ticagrelor (*N* = 570)		Prasugrel (*N* = 501)	
	Mean (SD) or *N* (%)	Mean (SD) or *N* (%)	*p* ^*∗*^	Mean (SD) or *N* (%)	*p* ^*∗*^
Adherence, mean PDC	89.3 (22.3)	89.4 (21.0)	0.06	88.1 (22.5)	0.26
Adherence, PDC >80%	11914 (82.7)	462 (81.3)	0.40	395 (78.8)	0.03
Switched drug	289 (2.0)	109 (19.2)	<0.001	64 (12.8)	<0.001
Persistence	10671 (74.1)	397 (69.9)	0.03	339 (67.7)	0.001

PDC, percent of days covered. ^*∗*^Each of the novel P2Y12 inhibitors was compared to clopidogrel, respectively.

**Table 4:** One-year rates of adverse events.

	Clopidogrel, *n* (%)	Ticagrelor, *n* (%)	*p* ^*∗*^	Prasugrel, *n* (%)	*p* ^*∗*^
Mortality	622 (4.3)	12 (2.1)	0.01	18 (3.6)	0.44
Myocardial infarction	817 (5.7)	31 (5.5)	0.80	22 (4.4)	0.23
Stroke	399 (2.8)	12 (2.1)	0.33	7 (1.4)	0.07
Bleeding events	519 (3.6)	17 (3.0)	0.43	14 (2.8)	0.33

^*∗*^Evaluated using the log-rank test with comparison group of clopidogrel.

**Table 5:** One-year rates of adverse events in propensity matched population.

	Clopidogrel		Ticagrelor		*p* ^*∗*^	Clopidogrel		Prasugrel		*p* ^*∗*^
	(*N* = 1704)		(*N* = 570)			(*n* = 1503)		(*n* = 501)		
Mortality	47	2.8	12	2.1	0.41	37	2.5	18	3.6	0.18
MI	75	4.4	31	5.5	0.31	42	2.8	22	4.4	0.08
Stroke	22	1.3	12	2.1	0.16	22	1.5	7	1.4	0.93
Bleeding events	33	1.9	17	3.0	0.14	33	2.2	14	2.8	0.44

^*∗*^Evaluated using the log-rank test with comparison group of clopidogrel.

**Table 6:** Hazard ratio (HR) and 95 confidence interval (CI) comparing the effect of novel P2Y12 inhibitors (ticagrelor or prasugrel) to clopidogrel on adverse events at one-year follow-up.

	Ticagrelor vs clopidogrel, HR (95% CI)			Prasugrel vs clopidogrel, HR (95% CI)		
	Unadjusted	Multivariable adjusted^*∗*^	Propensity adjusted^*∗∗*^	Unadjusted	Multivariable adjusted^*∗*^	Propensity adjusted^*∗∗*^
Mortality	0.49 (0.27–0.86)	0.67 (0.38–1.19)	0.43 (0.20–0.92)	0.83 (0.52–1.33)	1.54 (0.96–2.48)	1.42 (0.76–2.68)
Myocardial infarction	0.96 (0.67–1.37)	1.26 (0.87–1.81)	1.07 (0.68–1.68)	0.77 (0.51–1.18)	1.02 (0.67–1.57)	1.38 (0.81–2.34)
Stroke	0.75 (0.43–1.34)	1.19 (0.67–2.12)	1.52 (0.72–3.24)	0.50 (0.24–1.06)	0.94 (0.44–2.0)	0.94 (0.38–2.34)
Bleeding events	0.82 (0.51–1.34)	1.38 (0.85–2.25)	1.77 (0.94–3.31)	0.77 (0.45–1.31)	1.30 (0.76–2.23)	1.45 (0.75–2.80)

^*∗*^Multivariable proportional hazard (PH) regression models were developed for each outcome. For all outcomes, we controlled for demographics (age, gender, and race/ethnicity), precise DAPT score, medication adherence rate, creatinine, and hemoglobin. Additionally, for mortality, we controlled for prior myocardial infraction (MI), peripheral vascular disease, heart failure, diabetes, and chronic lung disease; for hospitalized MI, we controlled for prior MI, heart failure, diabetes, PCI indication, and anticoagulation use; for hospitalized stroke, we controlled for cerebrovascular disease, peripheral vascular disease, heart failure, diabetes, and anticoagulation use; for bleeding events, we controlled for peripheral vascular disease, heart failure, and anticoagulation. ^*∗∗*^Baseline variables in propensity score (PS) matching include demographics (age, gender, and race/ethnicity), cardiovascular history (prior MI, prior CABG, cerebrovascular disease, peripheral vascular disease, heart failure, atrial fibrillation, hyperlipidemia, hypertension, and diabetes), smoking status, chronic kidney disease, chronic lung disease, and PCI indication. For the subsequent multivariable models, all variables in the PS models were included, with additional variables including race/ethnicity, creatinine, hemoglobin, and medication adherence rate for all outcomes. We also adjusted anticoagulation for all hospitalized events, and precise DAPT score for mortality, MI, and bleeding events.

## Data Availability

The data used to support the findings of this study are available from the corresponding author upon request.

## Supplementary Materials

Supplementary Materials Supplementary Figure 1. Kaplan–Meier curves for clopidogrel, prasugrel, and ticagrelor for all measured outcomes. Supplementary Table 1(a). Baseline characteristics of excluded clopidogrel patients after matching with ticagrelor. Supplementary Table 1(b). Baseline characteristics of excluded clopidogrel patients after matching with prasugrel.

## References

[1] Wallentin L., Becker R. C., Budaj A. (2009). Ticagrelor versus clopidogrel in patients with acute coronary syndromes. *New England Journal of Medicine*.

[2] Wiviott S. D., Braunwald E., McCabe C. H. (2007). Prasugrel versus clopidogrel in patients with acute coronary syndromes. *New England Journal of Medicine*.

[3] You S. C., Rho Y., Bikdeli B. (2020). Association of ticagrelor vs clopidogrel with net adverse clinical events in patients with acute coronary syndrome undergoing percutaneous coronary intervention. *Journal of the American Medical Association*.

[4] Turgeon R. D., Koshman S. L., Youngson E. (2020). Association of ticagrelor vs clopidogrel with major adverse coronary events in patients with acute coronary syndrome undergoing percutaneous coronary intervention. *Journal of the American Medical Association Internal Medicine*.

[5] Ge Z., Baber U., Claessen B. E. (2019). Associations between use of prasugrel vs clopidogrel and outcomes by type of acute coronary syndrome: an analysis from the PROMETHEUS registry. *Journal of Thrombosis and Thrombolysis*.

[6] Gordon N. P. (2017). *Sociodemographic and Health-Related Characteristics of Members in Kaiser Permanente’s Northern California Region*.

[7] Brindis R. G., Fitzgerald S., Anderson H. V., Shaw R. E., Weintraub W. S., Williams J. F. (2001). The American College of cardiology—national cardiovascular data registry (ACC-NCDR): building a national clinical data repository. *Journal of the American College of Cardiology*.

[8] Freeman J. V., Reynolds K., Fang M. (2015). Digoxin and risk of death in adults with atrial fibrillation: the ATRIA-CVRN study. *Circulation: Arrhythmia and Electrophysiology*.

[9] Costa F., van Klaveren D., James S. (2017). PRECISE-DAPT Study Investigators. Derivation and validation of the predicting bleeding complications in patients undergoing stent implantation and subsequent dual antiplatelet therapy (PRECISE-DAPT) score: a pooled analysis of individual-patient datasets from clinical trials. *Lancet*.

[10] Sachdeva A., Mutyala R., Solomon M. D., Mcnulty E. J., Zhu S., Mantri N. (2022). A-47 | P2Y12 inhibitors in acute coronary syndromes: a real-world, community-based comparison of ischemic and bleeding outcomes. *Journal of the Society for Cardiovascular Angiography & Interventions*.

[11] Krishnamurthy A., Keeble C., Anderson M. (2019). Real-world comparison of clopidogrel, prasugrel and ticagrelor in patients undergoing primary percutaneous coronary intervention. *Open Heart*.

[12] Vercellino M., Sanchez F. A., Boasi V. (2017). Ticagrelor versus clopidogrel in real-world patients with ST elevation myocardial infarction: 1-year results by propensity score analysis. *BMC Cardiovascular Disorders*.

[13] Volz S., Petursson P., Odenstedt J. (2020). Ticagrelor is not superior to clopidogrel in patients with acute coronary syndromes undergoing PCI: a report from Swedish Coronary Angiography and Angioplasty Registry. *Journal of the American Heart Association*.

[14] Sahlen A., Varenhorst C., Lagerqvist B. (2016). Outcomes in patients treated with ticagrelor or clopidogrel after acute myocardial infarction: experiences from SWEDEHEART registry. *European Heart Journal*.

[15] Kim K., Lee T. A., Touchette D. R., DiDomenico R. J., Ardati A. K., Walton S. M. (2018). Comparison of 6-month costs between oral antiplatelet agents following acute coronary syndrome. *Journal of Managed Care & Specialty Pharmacy*.

[16] Capranzano P., Capodanno D. (2019). Switching between P2Y12 inhibitors: rationale, methods, and expected consequences. *Vascular Pharmacology*.

[17] Dayoub E. J., Seigerman M., Tuteja S. (2018). Trends in platelet adenosine d receptor inhibitor use and adherence among antiplatelet-naive patients after percutaneous coronary intervention, 2008-2016. *Journal of the American Medical Association Internal Medicine*.

[18] Mehran R., Baber U., Steg P. G. (2013). Cessation of dual antiplatelet treatment and cardiac events after percutaneous coronary intervention (PARIS): 2 year results from a prospective observational study. *The Lancet*.

[19] Lau H. S., de Boer A., Beuning K. S., Porsius A. (1997). Validation of pharmacy records in drug exposure assessment. *Journal of Clinical Epidemiology*.

