# Supplementary material for: P2Y12 Inhibitors in Acute Coronary Syndromes: A Real-World, Community-Based Comparison of Ischemic and Bleeding Outcomes
Source: J Interv Cardiol. 2023 May 20;2023:1147352. doi: 10.1155/2023/1147352 (PMC10224789; doi:10.1155/2023/1147352)

Supplementary Figure 1. Kaplan-Meier curves for clopidogrel, prasugrel, and ticagrelor for all measured outcomes.

1. Death


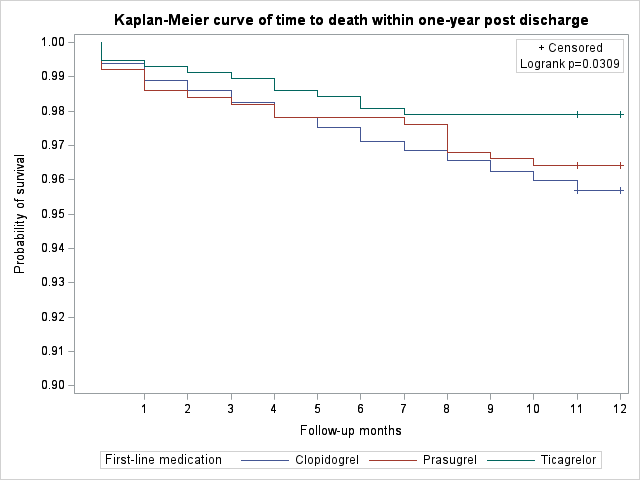


1. Myocardial Infarction


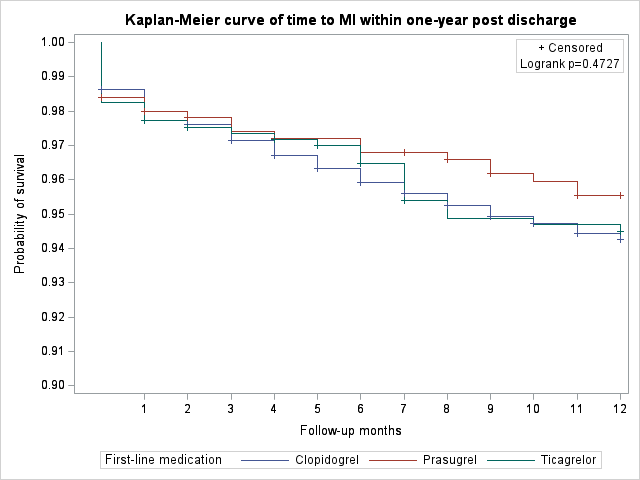


1. Stroke


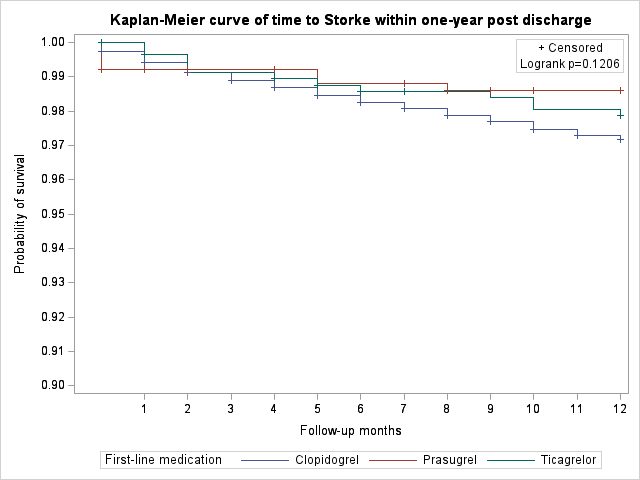


1. Bleeding


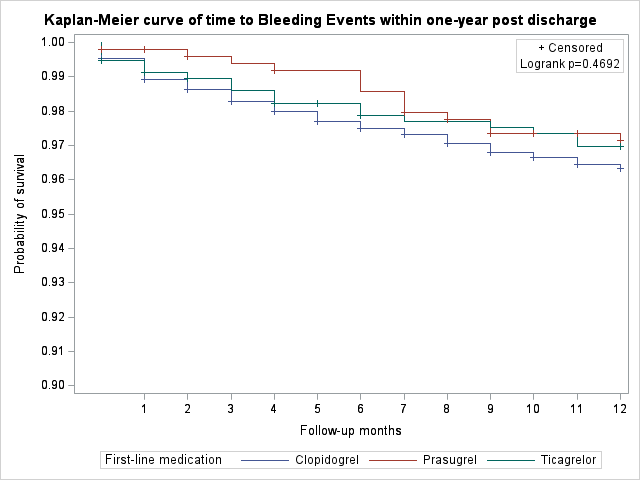

Supplement: Supplementary Materials — Supplementary Figure 1. Kaplan–Meier curves for clopidogrel, prasugrel, and ticagrelor for all measured outcomes. Supplementary Table 1(a). Baseline characteristics of excluded clopidogrel patients after matching with ticagrelor. Supplementary Table 1(b). Baseline characteristics of excluded clopidogrel patients after matching with prasugrel. [file 1147352.f1.zip › supplementary figure 1 (1).docx]
