# Supplementary material for: P2Y12 Inhibitors in Acute Coronary Syndromes: A Real-World, Community-Based Comparison of Ischemic and Bleeding Outcomes
Source: J Interv Cardiol. 2023 May 20;2023:1147352. doi: 10.1155/2023/1147352 (PMC10224789; doi:10.1155/2023/1147352)
Supplement: Supplementary Materials — Supplementary Figure 1. Kaplan–Meier curves for clopidogrel, prasugrel, and ticagrelor for all measured outcomes. Supplementary Table 1(a). Baseline characteristics of excluded clopidogrel patients after matching with ticagrelor. Supplementary Table 1(b). Baseline characteristics of excluded clopidogrel patients after matching with prasugrel. [file 1147352.f1.zip › supplementary table 1b.docx]

Supplementary Table 1(b) Baseline characteristics of excluded clopidogrel patients after matching with prasugrel.

| **Characteristics** | **Clopidogrel** | |
| --- | --- | --- |
|  | (n=12904) |  |
|  |  |  |
| **Demographics** |  |  |
| **Age** | 67.2±12.0 |  |
| **Female gender** | 3807 (29.5) | |
| **Race** |  |  |
| **White** | 8648 (67.0) | |
| **Black** | 781 (6.1) |  |
| **Asian** | 2249 (17.4) | |
| **Other/Unknown** | 1226 (9.5) |  |
| **Hispanic ethnicity** | 1623 (12.6) | |
| **Cardiovascular history** |  |  |
| **Prior MI** | 2904 (22.5) | |
| **Prior CABG** | 1544 (12.0) | |
| **Cerebrovascular disease** | 1199 (9.3) |  |
| **Peripheral vascular disease** | 5257 (40.7) | |
| **Heart failure** | 2939 (22.8) | |
| **Atrial fibrillation** | 1561 (12.1) | |
| **Hyperlipidemia** | 9093 (70.5) | |
| **Hypertension** | 9888 (76.6) | |
| **Diabetes** | 4688 (36.3) | |
|  |  |  |
| **Other risk factors** |  |  |
| **Smoking** |  |  |
| **Previous** | 5139 (39.8) | |
| **Current** | 1576 (12.2) | |
| **No/unknown** | 6189 (48.0) | |
| **Chronic kidney disease** | 3421 (26.5) | |
| **Chronic lung disease** | 1334 (10.3) | |
| **Dialysis** | 388 (3.0) |  |
| **Dyslipidemia** | 10317 (80.0) | |
| **Family history of premature CAD** | 1658 (12.8) | |
| **Liver disease** | 1009 (7.8) |  |
|  |  |  |
| **Laboratory data** |  |  |
| **Creatinine** | 1.1±1.1 |  |
| **Hemoglobin** | 13.3±1.9 |  |
| **Platelets** | 215.3±68.6 | |
| **Sodium** | 138.3±3.2 |  |
| **WBC count** | 8.6±3.7 |  |
|  |  |  |
| **Procedure details** |  |  |
| **PCI Indication** |  |  |
| **NSTEMI/unstable angina** | 10450 (81.0) | |
| **STEMI** | 2454 (19.0) | |
| **Bare metal stent** | 632 (4.9) |  |
| **Drug eluting stent** | 11760 (91.1) | |
| **Thrombectomy** | 711 (5.5) |  |
| **Bifurcation lesion** | 2814 (21.8) | |
| **Lesion complexity** |  |  |
| **High/C** | 6834 (53.0) | |
| **Non-High/non-C** | 6070 (47.0) | |
| **Previously treated lesion** | 682 (5.3) |  |
| **Vein graft PCI** | 562 (4.4) |  |
| **Cardiac arrest within 24 hours** | 196 (1.5) |  |
| **Cardiomyopathy or LV systolic dysfunction** | 1579 (12.2) | |
| **Cardiogenic shock within 24 hours** | 186 (1.6) |  |
| **Stress test** | 3198 (24.8) | |
| **Arterial access site** |  |  |
| **Brachial** | 15 (0.1) |  |
| **Femoral** | 7288 (56.5) | |
| **Radial** | 5596 (43.4) | |
| **Other** | 5 (0.0) |  |
| **Fluoroscopy time** | 17.8±13.2 |  |
| **Contrast volume (ml)** | 178.7±82.1 | |
| **Lesion length** | 29.3±21.3 |  |
|  |  |  |
| **Risk score** |  |  |
| **Precise DAPT score** | 28.5±18.7 |  |
|  |  |  |
| **Medication use post PCI** |  |  |
| **ACE inhibitors** | 7844 (60.8) | |
| **ARBs** | 3374 (26.1) | |
| **Oral Anticoagulation** | 1451 (11.2) | |
| **Beta blockers** | 12095 (93.7) | |
| **Statins** | 12499 (96.9) | |
